# Characterization and Discrimination of Ophiopogonis Radix with Different Levels of Sulfur Fumigation Based on UPLC-QTOF-MS Combined Molecular Networking with Multivariate Statistical Analysis

**DOI:** 10.3390/metabo13020204

**Published:** 2023-01-30

**Authors:** Yanhui Lv, Xike Xu, Yanping Wei, Yunheng Shen, Wei Chen, Xintong Wei, Jie Wang, Jiayun Xin, Jixiang He, Xianpeng Zu

**Affiliations:** 1 School of Pharmacy, Shandong University of Traditional Chinese Medicine, Jinan 250355, China; 2 School of Pharmacy, Naval Medical University, Shanghai 200433, China

**Keywords:** Ophiopogonis Radix, sulfur fumigation, UPLC-QTOF-MS, molecular networking, multivariate statistical analysis

## Abstract

Ophiopogonis Radix, also known as “Maidong” (MD) in China, is frequently sulfur-fumigated (SF) in the pretreatment process of MD to improve the appearance and facilitate preservation. However, the process leads to changes in chemical composition, so it is essential to develop an approach to identify the chemical characteristics between nonfumigated and sulfur-fumigated products. This paper provided a practical method based on UPLC-QTOF-MS combined Global Natural Products Social Molecular Networking (GNPS) with multivariate statistical analysis for the characterization and discrimination of MD with different levels of sulfur fumigation, high concentration sulfur fumigation (HS), low concentration sulfur fumigation (LS) and without sulfur fumigation (WS). First, a number of 98 compounds were identified in those MD samples. Additionally, the results of Principal component analysis (PCA) and Orthogonal partial least-squares-discriminant analysis (OPLS-DA) demonstrated that there were significant chemical differences in the chemical composition of MD with different degrees of SF. Finally, fourteen and sixteen chemical markers were identified upon the comparison between HS and WS, LS and WS, respectively. Overall, these results can be able to discriminate MD with different levels of SF as well as establish a solid foundation for further quality control and pharmacological research.

## 1. Introduction

Generally, all herbs transform the raw materials into a usable form through processing for better effects [1]. As one of the processing methods, sulfur fumigation (SF) has various functions such as insect control, mildew prevention, sterilization, moisture prevention, etc. which is also beneficial for the storage and beautification of the appearance and color of herbs [2,3]. Accumulating investigations indicated that sulfur fumigation leads to excessive sulfur dioxide residues and even causes changes in the chemical composition of herbs, which not only affects the quality of herbs but also presents a safety risk [4,5,6]. Therefore, it is of great importance to ascertain whether the chemical constituents of a sulfur-fumigated herb changed or not. And whether and how sulfur fumigation affects the whole quality of sulfur-fumigated herbs requires further investigation.

Ophiopogonis Radix (called “Maidong” in China), the tuberous root of *Ophiopogon japonicus* (Thunb.) Ker-Gawl, belongs to the Liliaceae family and is commonly applied as traditional Chinese medicine (TCM) documented in the clinical. Maidong (MD) was first mentioned in Variorum of Shennong’s Classic of Materia Medica (Shennong Bencao Jing) with the effect of nourishing yin and generating body fluid, moistening the lung and clearing the heart [7,8]. Additionally, modern phytochemical studies have suggested that MD is rich in various compounds, including steroidal saponins, homoisoflavonoids, and polysaccharides, which exhibit cardiovascular protection, anti-cancer, immunomodulatory, anti-inflammatory, central nervous system protective, antioxidative, and anti-diabetes effects [9,10,11,12,13]. Thus, the quality of MD will directly affect its safety and effectiveness. In order to obtain a better appearance, sulfur fumigation is frequently employed in the processing of fresh MD, which usually contributes to excessive sulfur fumigation [14]. A report demonstrated that homoisoflavonoids would undergo sulfation and sulphite esterification during sulfur fumigation [15,16]. Therefore, it is crucial to determine the differences in chemical compositions between MD with different levels of SF, high concentration sulfur fumigation (HS), low concentration sulfur fumigation (LS) and without sulfur fumigation (WS).

Ultra-performance liquid chromatography coupled with time-of-flight mass spectrometry (UPLC-QTOF-MS) is a promising platform, which is widely used to identify the chemical components of herbs due to its high sensitivity and resolution [17,18,19]. Global natural products social molecular networking (GNPS, https://gnps.ucsd.edu, accessed on 7 November 2022) is a public data-sharing web platform that can systematically compare and classify thousands of molecules based on the similarity of MS/MS spectra and can visualize the relationship between these molecules. At present, GNPS is widely used in metabolomics, the identification of chemical components of herbal medicines and discovery of new compounds [20,21,22,23]. Additionally, multivariate statistical analysis, including principal component analysis (PCA) and orthogonal partial least-squares-discriminant analysis (OPLS-DA), provides a credible way to evaluate the holistic variation, and identify chemical markers in distinguishing origin, different parts and processing methods of herbs [24,25,26,27].

In this work, we established a new method by combining UPLC-QTOF-MS with the MN method to identify the chemical compounds of MD samples. Furthermore, the combination of multivariate statistical analysis with heatmap analysis was used to evaluate the holistic discrepancy and explore the chemical markers of MD with different levels of SF. This work would be useful for the characterization and discrimination of Ophiopogonis Radix with different levels of SF, hoping to provide a theoretical basis for further research on the pharmacological effect, quality evaluation, and clinical application of MD. The proposed approach may also provide a feasible way to investigate of chemical components and potential chemical markers in other herbs.

## 2. Materials and Methods

### 2.1. Samples and Chemical Reagents

A total of 15 batches of Ophiopogonis Radix, derived from 3 different levels of sulfur fumigation, were purchased from herbal markets. The herb of Ophiopogonis Radix was confirmed by Prof. Bao-Kang Huang (Department of Pharmacognosy, Naval Medical University, Shanghai, China). The Department of Natural Medicinal Chemistry, School of Pharmacy, Naval Medical University, Shanghai, China maintained the Voucher specimens.

Methanol, acetonitrile, formic acid, and water (LC-MS-grade) were purchased from Fisher Scientific Co. (Fair Lawn, NJ, USA); leucine enkephalin was provided by Sigma-Aldrich (St. Louis, MO, USA).

### 2.2. Sulfur-Fumigated MD Samples

All MD samples were randomly divided into WS, LS, and HS groups, and the amount of sulfur fumigation in the three groups was 0 g, 10 g, and 20 g, respectively. 50 g of MD herbs were wetted with 5 mL water, then the sulfur was burned and put into the bottom of the desiccator, and the herbs were placed in the upper layer of the desiccator and fumigated airtight for 24 h. After fumigation, the sample of MD was dried in the oven at 50 °C for 12 h [15].

### 2.3. Sample Preparation

The MD herbs were crushed to produce the powder. Then 100 mg powder from each sample was accurately weighed into a 2 mL centrifuge tube and soaked in 1 mL of 80% methanol overnight to maintain a stable concentration. The next day, the extract was ultrasonic-extracted for 60 min at room temperature and then centrifuged for 10 min at 12,000 rpm. The supernatant was filtered through a 0.22 μm syringe filter and the renewed filtrate was transferred to a 1.5 mL centrifuge tube and stored at 4 °C prior to the analysis.

### 2.4. Chromatographic and MS Spectrometry Conditions

Chromatographic analysis was performed with an ACQUITY UPLC I-class system (Waters, Milford, MA, USA) equipped with a binary solvent delivery system, an autosampler, a degasser and a thermostat column compartment. An ACQUITY HSS T3 column (2.1 mm × 100 mm, 1.8 μm, Waters, Milford, MA, USA) was chosen for chromatographic separations. The mobile phase consisted of solvent A (0.1% formic acid in water) and solvent B (acetonitrile) with the following gradient elution program: 0–0.5 min, 5%B; 0.5–1.5 min, 5–40%B; 1.5–13 min, 40–80%B; 13–20 min, 80–84%B; 20–23 min, 84–95%B; 23–23.5 min, 95–5%B; 23.5–25 min, 5%B. The injection volume was set at 1 μL. Flow rate was 0.4 mL/min.

Mass spectrometric analysis was performed on a Waters SYNAPT G2-Si HRMS system with a ZSpray™ electrospray ion trap (ESI) source. The desolvation gas flow rate and cone gas flow rate were 800 L/h and 50 L/h, respectively; source offset voltage, 80 V; cone voltage, 40 V; and the source temperature was set to 120 °C; the capillary voltage, 2.5 kV. MS^E^ and fast-DDA modes of data acquisition were used. In the MS^E^ mode, the acquired mass spectra range was set from 50 to 1500 Da, scan time, 0.3 s, and the high collision energy was 10−45 eV, low collision energy was 6 eV. MS/MS fragments of all precursor ions can be produced in MS^E^ mode, which was used for multivariable statistical analysis. The energy of the dual dynamic collision in fast DDA mode was 6–40 V (50 Da) and 40−120 V (1500 Da). The MS and MS/MS scan rate was 0.2 s. The top 5 strong ions of the precursor ions were selected for MS/MS fragmentation [28], and these data-dependent type data were able to establish MN. Leucine encephalin solution (*m/z* 554.2615 [M-H]^−^) was used as a reference to calibrate the data in real-time. Data acquisition was performed by MassLynx v4.1 software (Waters, Milford, MA, USA).

### 2.5. Molecular Networking

The UPLC-QTOF-MS-MS raw data was converted into “mzXML” format by MSConvert (http://proteowizard.sourceforge.net, accessed on 5 November 2022) and then uploaded to the GNPS online platform [29]. The GNPS parameters were set as follows: Both the precursor ion mass tolerance and the fragment ion tolerance were set at 0.02 Da in this work. The cosine score with a value of more than 0.7, meanwhile, there were at least six matched peaks. A molecular network was created, and then a visualization was performed by Cytoscape 3.9.1. (http://www.cytoscape.org/, accessed on 10 November 2022).

### 2.6. Multivariate Statistical Analysis

The combination of UPLC-QTOF-MS/MS technology with multivariate statistical analysis could not only characterize chemical constituents but also rapidly detect potential chemical markers [30,31]. The raw data of mass spectrometry were preprocessed by One-Map (http://www.5omics.com/, accessed on 1 November 2022). All preprocessed LC-MS data were imported into SIMCA-P 14.1 software (Umetrics, Sweden) for the multivariate statistical analysis. Principal component analysis (PCA) and orthogonal partial least-squares-discriminant analysis (OPLS-DA) were carried out to evaluate the relationship among experimental groups. PCA, an unsupervised analysis method, can convert the original variables into new irrelevant variables, combining duplicate information. Unlike the PCA model, OPLS-DA is a supervised model which filters systematic noise and extracts variable information. Further, in order to obtain chemical markers S-plots, volcano plots and variable importance for the projection (VIP) value were performed. The volcano plots were generated using GraphPad Prime 8.0. Based on the chemical markers identified by VIP value > 1, *p*-value < 0.05 (Independent-samples *t*-test) and fold changes (FC) > 2.0 or <0.8, the heatmap and correlation analysis were performed by MetaboAnalyst 5.0 (https://www.metaboanalyst.ca/, accessed on 15 October 2022). And the correlation between the two variables is denoted by the letter *r* and quantified by a number from −1 to +1. The direction of the correlation is indicated by the sign of the *r*. There is no correlation if *r* is zero, and the closer *r* is to 1, the more correlated the two compounds are [32,33].

## 3. Results and Discussion

### 3.1. Identification of Chemical Constituents in MD with Different Levels of SF

**Figure 1:**
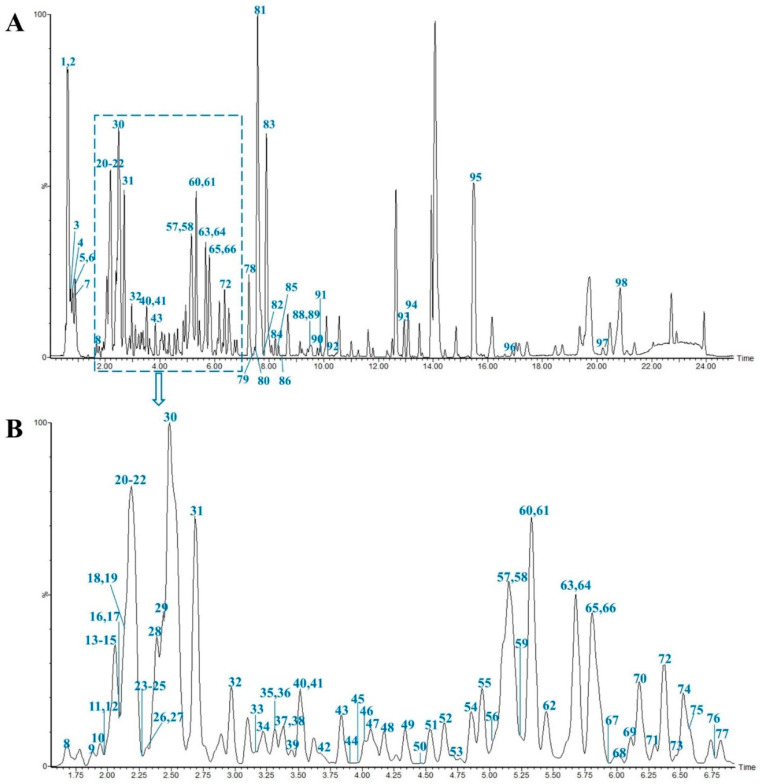
Representative base peak intensity chromatogram of Ophiopogonis Radix in the negative ion mode. The main chromatogram (**A**) and its enlargement (**B**).

In this study, all the compounds were determined from MS data combined with the MN chemical composition database and relevant literature. As a result, a total of 98 compounds including 28 steroidal saponins, 40 homoisoflavonoids, 8 organic acids, 4 alkaloids, 3 polysaccharides and 15 others were identified or tentatively characterized. The compounds were identified according to the established method of analysis based on retention times (Rt), fragment ions and fragment pathways referring to the literature information. And the representative base peak intensity chromatogram (BPI) of MD in negative ion mode is shown in Figure 1. As we can see from the Figure 1, peak **1**, **2**, **30**, **81** and **83** have high mass spectrometry responses, so they are relatively easy to research, and they are identified as Stachyose, Raffinose, Cryptomeridiol-11-O-β-D-xylopyranosyl-(1-6)-β-D-glucopyranoside, Methylophiopogonanone A, Methylophiopogonanone B, respectively. Detailed information on the components, including peak number, Rt, compound names, formulas, classifications, mass errors and fragment ions, are shown in Table S1.

**Figure 2:**
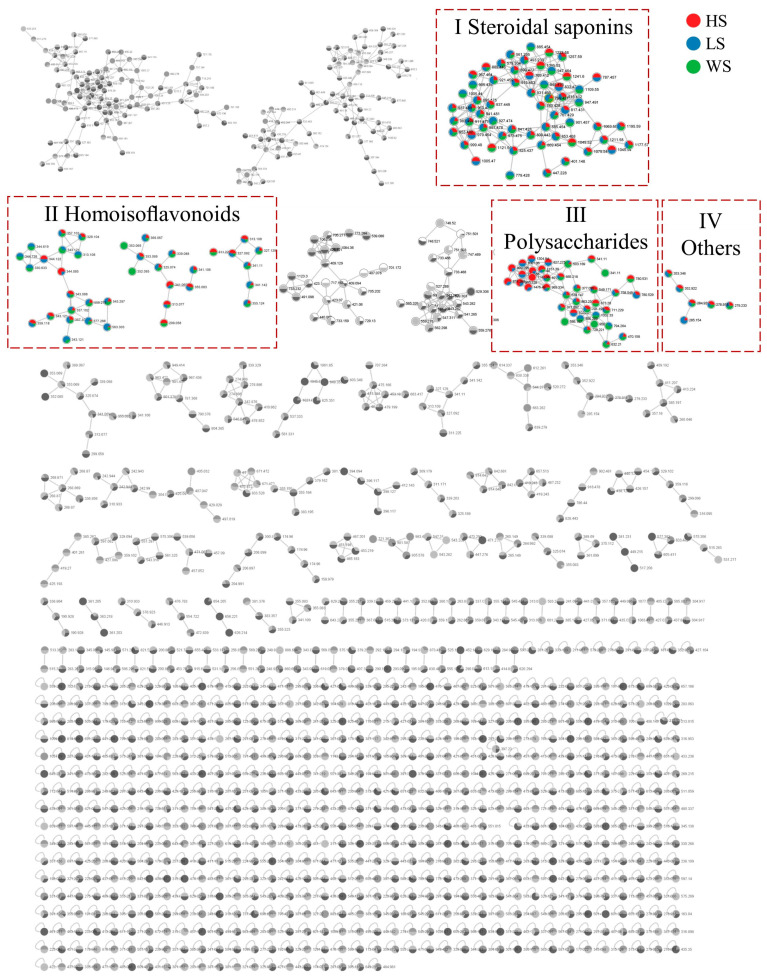
The molecular networks of MD with different levels of SF. (Ⅰ) Steroidal saponins; (Ⅱ) Homoisoflavonoids; (Ⅲ) Polysaccharide; (IV) Others. HS: high concentration sulfur fumigation; LS: low concentration sulfur fumigation; WS: without sulfur fumigation.

In this paper, the integrative molecular network of MD with different levels of SF was obtained on the basis of their MS/MS spectral similarity, as shown in Figure 2. The molecular network was composed of 1285 precursor ions, which included 101 clusters (nodes ≥ 2) and 622 single nodes. More details can be found on the GNPS website (https://gnps.ucsd.edu/ProteoSAFe/status.jsp?task=4da2a1e22a1943b9bf0fc3643f75a805, accessed on 7 November 2022). The proportion of the red, blue and green sectors at each node in MN represents the relative content of the compound in HS, LS and WS, respectively. In molecular networking, compounds with similar structures tend to cluster together in order to facilitate analysis [34]. It can be found that steroidal saponins (I), homoisoflavonoids (Ⅱ), polysaccharides (Ⅲ) and other compounds (Ⅳ) can form different clusters in the molecular networking. The compounds were identified according to established method of analysis based on Rt, the GNPS library and fragment pathway referring to the literature information. Representative compounds of each cluster are shown in Figure S1.

#### 3.1.1. Identify Elucidation of Steroidal Saponins

Cluster I mainly contained the nodes of steroidal saponins. Steroidal saponins are an important class of natural products and exhibit a wide range of pharmacological effects. Steroidal saponins are the main representative components of MD. Most of the steroidal saponins isolated and identified from MD are spirostanol saponins, and a few are furostanol saponins. Tetrasaccharide glycosides and monoglycosides make up a smaller portion of steroidal saponins, which are primarily trisaccharide and disaccharide glycosides. Rhamnose, fucose, glucose, and xylose, along with a small amount of arabinose, are the major polysaccharides in the steroidal saponins. Although it is generally accepted that steroidal saponins can provide less information in the negative ion mode [35], we found that steroidal saponins have good signals in the negative mode and are capable of forming strong quasi-molecular ion peaks of [M-H]^−^ and [M+HCOO]^−^ in MS spectra, which is beneficial to determine the relative molecular mass and molecular formula of steroidal saponins. Generally, the characteristic losses of steroidal saponins were 162(-glucose), 146(-rhamnose or -fucose), 132(-xylose or -arabinose) and corresponding acetylated monose such as 188(Ac-rhamnose) and 174(Ac-xylose) [36].

**Figure 3:**
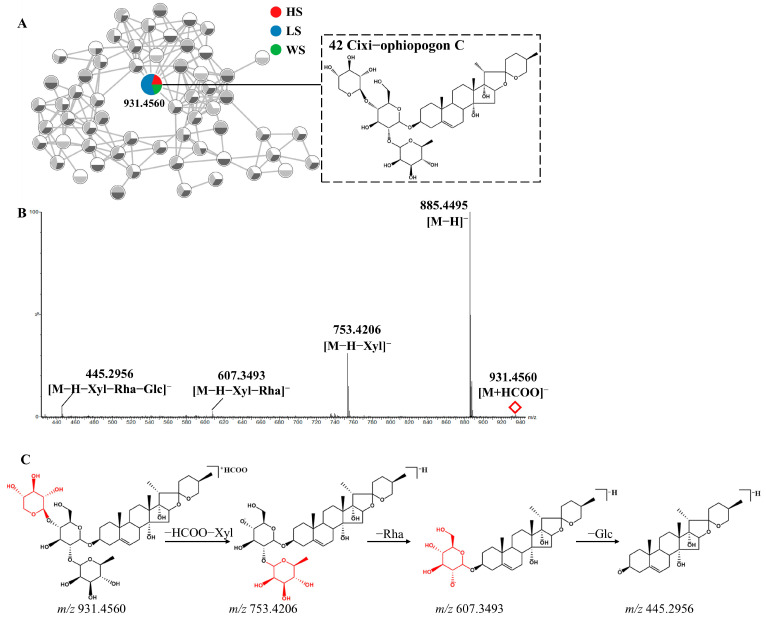
The enlargement of cluster I molecular network (**A**), MS/MS spectra (**B**) and possible fragmentation pathway(**C**) of Cixi-ophiopogon C (Compound **42**) from MD in negative ion mode.

Peak **42** (Rt = 3.65 min) was taken as an example to elucidate the detailed fragmentation pathway of steroidal saponins. Figure 3 displayed its MS/MS spectra and potential fragmentation pathway. Peak **42** exhibited the [M+HCOO]^−^ ion at *m/z* 931.4560 with molecular formula C_44_H_70_O_18_. Its MS/MS spectrum produced [M-H]^−^ ion at *m/z* 885.4495 and fragment ions at *m/z* 753.4206, 607.3493 and 445.2956, corresponding to [M-H-xylose]^−^, [M-H-xylose-rhamnose]^−^ and [M-H-xylose-rhamnose-glucose]^−^ respectively. Compared with the literature, peak **42** was tentatively characterized as Cixi−ophiopogon C [37]. Two isomers (peaks **73** and **77**) showed [M+HCOO]^−^ ions at *m/z* 899.4644 with the molecular formula C_44_H_70_O_16_, the fragment ions at *m/z* 721.4142 and 575.3587, resulting from the loss of a xylose and a successive loss of rhamnose. Based on the fragmentation pattern and the related literature, they were tentatively identified as Ophiopogonin D and Sprengerinin C, respectively [38,39].

With the aid of GNPS, a total of 28 steroidal saponins (9 disaccharide glycosides, 10 trisaccharide glycosides, 5 tetraglycoside and 4 pentaglycoside) were ambiguously or tentatively identified by comparing with the literature [40,41,42]. In addition, it is notable that there were some unidentified compounds in the BPI chromatogram and further investigation was necessary. The representative chemical structures of steroidal saponins are shown in Figure S1A.

#### 3.1.2. Identify Elucidation of Homoisoflavonoids

**Figure 4:**
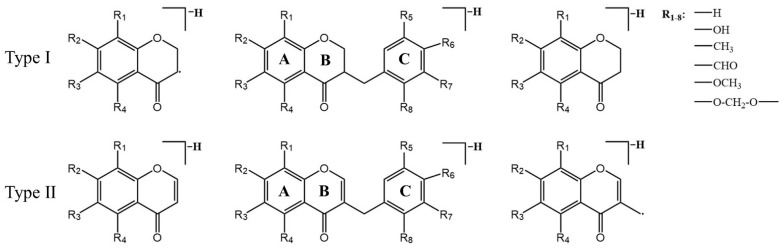
Proposed fragment ions from the predominant fragmentation of homoisoflavonoids.

Cluster II is homoisoflavonoids. Homoisoflavonoids, a particular class of flavonoids, are also the main chemical constituents of MD. Homoisoflavonoids exist in the form of aglycon, which has a basic skeleton with one more carbon atom than isoflavones. In total, 40 homoisoflavonoids were detected, which were mainly divided into 3-benzyl-4-chromanone (I) and 3-benzyl-4-chromone (II) types due to their nucleus structures (Figure 4). According to the previous report [43,44,45], the type I homoisoflavonoids cleavage pathway is usually through the cleavage of the C3-C9 bond with the simultaneous elimination of the benzyl portion of the B-ring. The type II homoisoflavonoids, containing an unsaturated C2-C3 bond, tended to lose B-ring on account of the cleavage of C9-C1′([M-H-B-ring]^−^) or C9-C3([M-H-B-ring-CH_2_+H]^−^) and yield two productions which usually showed a 13 Da (−CH_2_+H) characteristic mass difference.

**Figure 5:**
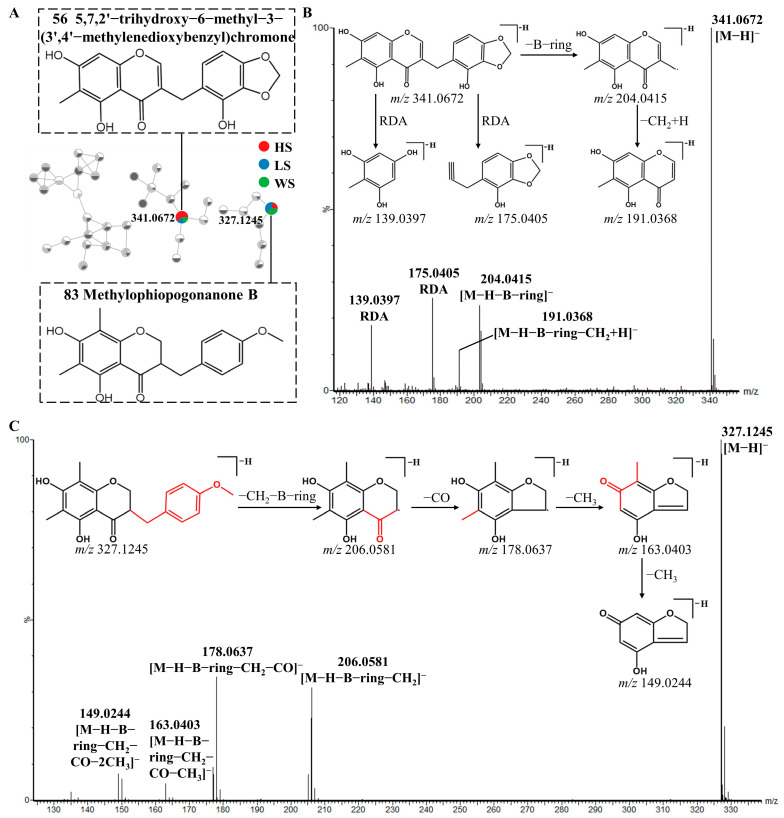
Possible fragmentation pathway of homoisoflavonoids: The enlargement of cluster Ⅲ molecular network (**A**), MS/MS spectra and fragmentation behaviors (**B**,**C**) of 5,7,2′-trihydroxy-6-methyl-3-(3′,4′-methylenedioxybenzyl) chromone (Compound **56**) and Methylophiopogonanone B (Compound **83**), respectively.

Peak **56** displayed a [M-H]^−^ ion at *m/z* 341.0672. The 13 Da characteristic mass difference from *m/z* 191.0368 to 204.0415 indicated that they were type II homoisoflavonoids, additionally peak **56** also underwent the retro-Diels-Alder (RDA) cleavage and yielded the product ions at *m/z* 175.0405 and 139.0397. Thus, peak **56** was tentatively identified as 5,7,2′-trihydroxy-6-methyl-3-(3′,4′-methylenedioxybenzyl) chromone (Figure 5B) based on the related literature [36]. The type I homoisoflavonoids fragmentation behavior was also observed in Peak **83**. Peak **83** showed [M-H]^−^ ion at *m/z* 327.1245 and generated fragments ions at *m/z* 206.0581([M-H-B-ring-CH_2_]^−^), which is the same as that reported in the literature [46]. In addition, the [(M-H-B-ring-CH_2_-CO]^−^ product ion was observed at *m/z* 178.0637. Besides the fragment ions reported in the literature, the product ions at *m/z* 163.0403, 149.0244, 135.0451 were also observed [42]. The fragment ion at *m/z* 163.0403 was derived from a neutral loss of CH_3_ by the fragment ion at *m/z* 178.0637. The *m/z* 163.0403 ion further fragmented to produce fragment ions at *m/z* 149.0244 and 135.0451 by the neutral loss of CH_3_ and CO, respectively. As a result, peak **83** was assumed to be Methylophiopogonanone B (Figure 5C).

Based on the MN, forty kinds of homoisoflavonoids were identified or tentative identified, including 22 type Ⅰ homoisoflavonoids and 18 type II homoisoflavonoids. The representative chemical structures of steroidal saponins are shown in Figure S1B.

#### 3.1.3. Identify Elucidation of Polysaccharides

In this study, three polysaccharides (peaks **1**, **2**, **4**) were identified by MN in cluster Ⅲ. The main typical fragment ions of the polysaccharides were found by losing glucose (162 Da). Peak **2** was taken as an example to illustrate the fragmentation pathway for polysac-charides. It afforded an adduct ion at *m/z* 539.1371[M+Cl]^−^, the formula of which was calculated as C_18_H_32_O_16_. Its MS/MS spectrum yielded a series of product ions at *m/z* 503.1642, 383.1262, 341.1096, 323.1010, 221.0700, 179.0558, showing no difference with the literature results [47]. Therefore, the chemical structure of peak **2** is tentatively identified as Raffinose (Figure S2). And the other two polysaccharides (Stachyose and Fungitetraose) were also identified. The representative chemical structures of steroidal saponins are shown in Figure S1C.

#### 3.1.4. Identify Elucidation of Other Compounds

In the present work, with the exception of the steroidal saponins, homoisoflavonoids and polysaccharides mentioned above, 27 other compounds (peaks **3**, **5**, **6**, **7**, **8**, **9**, **10**, **12**, **14**, **15**, **16**, **20**, **25**, **28**, **29**, **30**, **31**, **34**, **45**, **75**, **85**, **86**, **94**, **95**, **96,** **97**, **98**) were putatively identi-fied by matching the fragment ions with those provided in the literature [48,49]. The structures of n-tricosanoic acid and (2aS,3aS)-lyciumamide D are shown in Figure S1D.

### 3.2. Discrimination of MD and Its Sulfur-Fumigated Products by Multivariate Statistical Analysis

**Figure 6:**
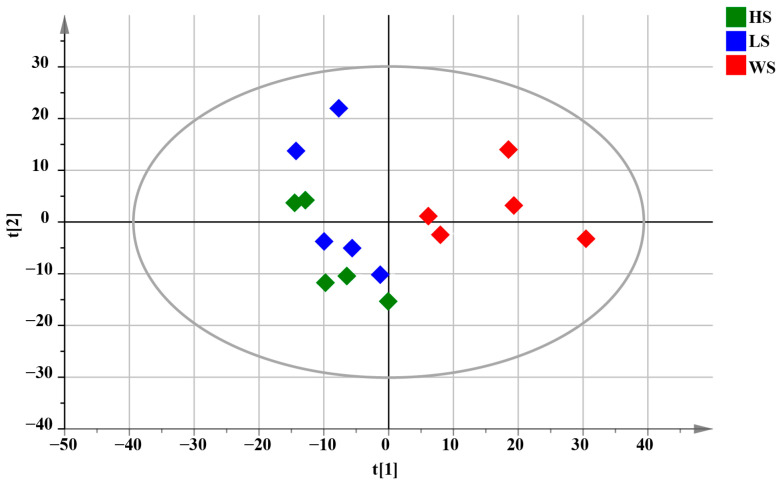
PCA score plots of total samples for MD with different levels of SF, R^2^X = 0.631.

Multivariate statistical methods were used to analyze the data to accurately reveal the chemical differences of MD with different levels of SF. PCA is capable of being used to reveal trends in the dataset [50]. Therefore, PCA analysis was performed to visualize the clustering trends among the HS, LS and WS groups. PCA results were shown in Figure 6, each dot in the PCA score plot represents a sample. From the preliminary PCA score plot, it could be visualized that MD with different degrees of SF showed a certain trend of separation, meaning that there were some differences in chemical composition. The WS group was farther away from the other two groups in the PCA score plots, indicating that the metabolic of WS had a greater difference from HS and LS.

In order to highlight the intergroup differences between HS, LS and WS, OPLS-DA was used to search for the chemical markers. Contrary to the PCA model, OPLS-DA is a supervised model which can maximize the difference between groups, and thus determine the chemically different components between groups [50]. Based on the PCA results (Figure S3A), two new OPLS-DA models were established and the group was as follows: HS versus WS; LS versus WS.

**Figure 7:**
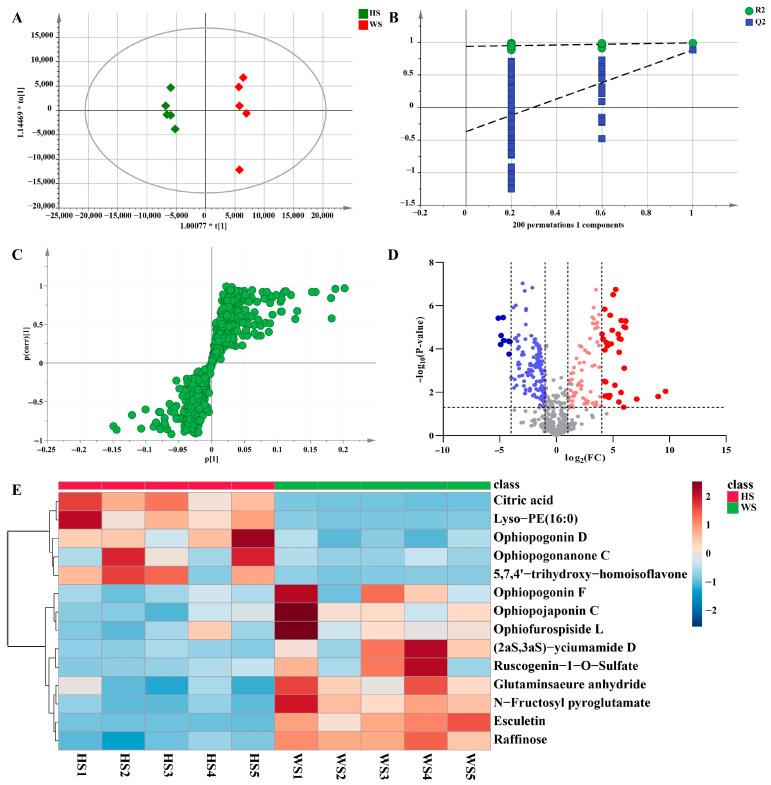
Multivariate statistical analysis result of HS and WS groups: OPLS-DA (**A**) score plots and OPLS-DA model permutation test (**B**), S-plot (**C**) and Volcano plots (**D**), (**C**,**D**) were used to explore markers in HS and WS, the heatmap of potential chemical markers (**E**). Lyso-PE (16:0): 1-hexadecanoyl-sn-glycero-3-phosphoethanolamine.

In the pairwise comparison of HS vs. WS, the statistical parameters of OPLS-DA R^2^X (cum), R^2^Y (cum), and Q2 (cum) were 0.621, 0.992, and 0.884, respectively, indicating that the model had good repeatability and predictability (Figure 7A). To validate the performance of the model, a 200-iteration permutation test was carried out in the OPLS-DA model. In the permutations plot, both Q^2^ and R^2^ (Q^2^ = [0.0, 0.939]; Q2 = [0.0, −0.369]) values were obviously lower than the relevant original values (Figure 7B). The results showed that the OPLS-DA model has no randomness and overfitting. Meanwhile, S-plots and volcano plots were created under OPLS-DA models to quickly and visually discover key markers (Figure 7C,D). In volcano plots, red spots represented variables of high abundance with significant differences, while green spots represented variables of low abundance. Compounds with VIP > 1, FC > 2 or < 0.8, and *p* < 0.05 from univariate statistical analysis were regarded as the chemical markers between MD samples. Finally, fourteen markers showing notable differences between HS and WS were identified (Table 1), in which these potential markers were visualized by a heat map (Figure 7E). The darker the color was, the stronger was the degree of increase or decrease in metabolite concentration. As we can see from the figure, there were six chemical markers (peak **6**, **37**, **73**, **85**, **86**, **90**) with higher relative contents in HS than in WS, however, nine chemical markers (peak **2**, **5**, **7**, **15**, **17**, **18**, **31**, **42**, **75**) were higher in WS than in HS. These results suggested that the contents of some of the compounds changed after SF.

**Figure 8:**
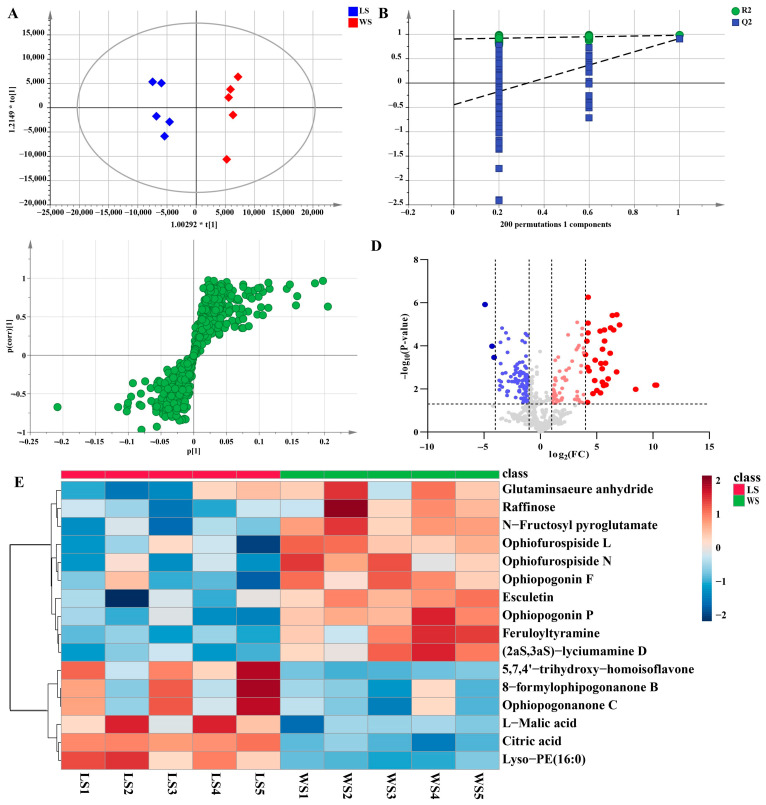
Multivariate statistical analysis result of LS and WS groups: OPLS-DA (**A**) score plots and OPLS-DA model permutation test (**B**), S-plot (**C**) and Volcano plots (**D**), (**C**,**D**) were used to explore markers in LS and WS, the heatmap of potential chemical markers (**E**).

Additionally, a pairwise comparison was performed for the LS and WS groups in the same way. In this study, LS and WS samples were clearly separated in the PCA scores plot (Figure S3B). The OPLS-DA score plot also further indicated a significant difference in the two groups that the model was a noticeably significant model (R^2^X = 0.683, R^2^Y = 0.981, Q^2^ = 0.914) (Figure 8A). The established OPLS-DA model (Figure 8B) was considered to be reliable and reproducible (R^2^ = [0.0, 0.905], Q^2^ = [0.0, −0.446]). Similarly, s-plots and volcano plots (Figure 8C,D) were created to identify chemical markers. In this work, based on VIP > 1, FC > 2 or < 0.8, and *p* < 0.05, 16 potential markers were detected as shown in Table 1. The results were shown intuitively in Figure 8E in the form of a heatmap. Compared with non-sulfur fumigated Ophiopogonis Radix, the content of homoisoflavonids was much higher after Sulfur fumigation, such as 5,7,4′-trihydroxy-homoisoflavone (**37**), Ophiopogonanone C (**90**), 8-formylophipogonanone B (**92**). However, the steroidal saponins content was completely contrary to homoisoflavonids, such as Ophiopogonin F (**18**), Ophiopogonin P (**13**), Ophiofurospiside L (**17**), etc.

According to our research, it is speculated that the increase in the content of compounds **37** and **90** is probably due to an additional reaction between unsaturated carbonyl groups in the structure with sulfurous acid [15]. It has been reported that in the process of SF, sulfur dioxide can combine with water and the sodium salts contained in the herb and eventually form sodium bisulfite. A substitution reaction then occurs between the bisulfite and the hemiacetal hydroxyl group on the sugar, which results in a reduction of the sugar content [51]. Coincidentally, we also found this result in our study, such as a decrease in the content of compound **2** after SF. The results of this study may help to elucidate the reactions that happened during the processing of SF. However, further research is needed to determine whether the components that have changed during the sulfur fumigation process will affect the pharmacological effects of MD.

### 3.3. Correlation Analysis of Chemical Markers

**Figure 9:**
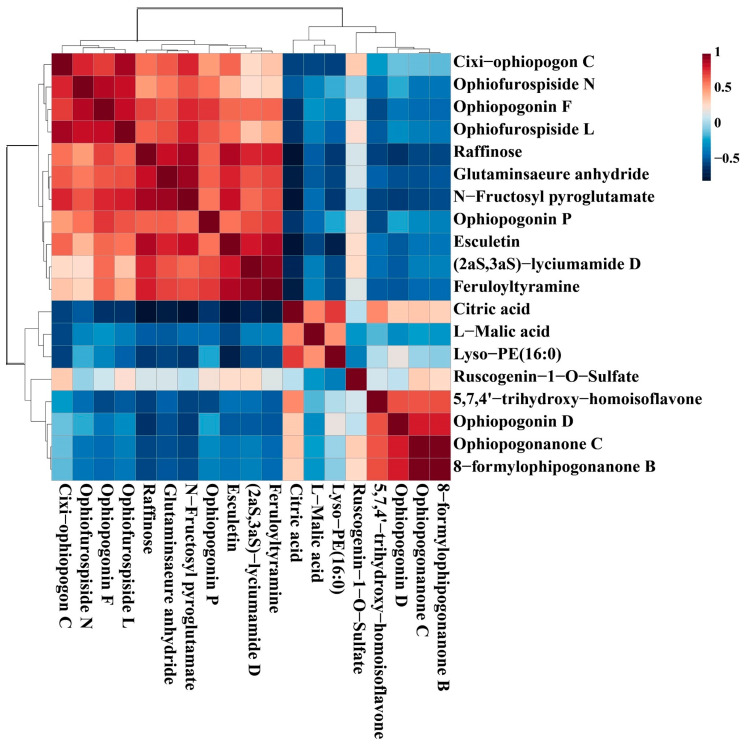
The correlation analysis of potential markers of MD with different levels of SF.

Correlation is defined as a relationship that exists between phenomena or things or between mathematical or statistical variables. In our work, the correlation analysis was performed to further analyze the relationship of the 19 chemical markers from different levels of SF. The color of each square in the figure represents the correlation of two markers: red denotes positive correlation, and blue denotes negative correlation; the darker the color, the more significant the correlation. As shown in Figure 9, only Citric acid and Lyso-PE (16:0) had positive correlations with L-Malic acid, which had negative correlations with the majority of the other compounds; Ophiopogonanone C and 8-formylophipogonanone B have a very strong positive correlation, indicating that their levels are changing in a consistent trend. The results indicated that the interaction of differential compounds of MD was obviously different. These chemical markers as a whole showed some correlation, suggesting that these compounds were likely to be related in biosynthesis.

## 4. Conclusions

In a word, a comprehensive method based on the combination of a multiple-dimensional collection model and various data analysis methods was developed for the characterization and classification of MD with different levels of SF. Based on GNPS, a total of 98 constituents, including 28 steroidal saponins, 40 homoisoflavonoids, 8 organic acids, 4 alkaloids, 3 polysaccharides, and 15 Others, were unambiguously identified or tentatively characterized in MD samples. Then the multivariate statistical analysis coupled with the heatmap showed an obvious separation between the MD with different levels of SF. Especially, 14 and 16 potential chemical markers were rapidly detected upon the comparison between HS and WS, LS and WS, respectively, which might be used for the classification of MD with different levels of SF. These results illustrated that our work provided a better method for the identification of the chemical components of MD and a viable strategy to authenticate MD with different levels of SF. The proposed approach was efficient to reveal SF effect on the herb chemical constituents. Further research is needed to determine the mechanism of the transformation of chemical constituents during SF and whether sulfur fumigation will affect the pharmacological effects of MD.

## Figures and Tables

**Table 1:** The VIP value, Fold change and *p*-value of chemical markers.

Group	No.	Compound Name (the Number in Table 1)	VIP Value	Fold Change	*p*-Value
HS vs. WS	1	Raffinose (**2**)	4.60	0.35	<0.001
	2	N-Fructosyl pyroglutamate (**5**)	2.95	0.09	<0.001
	3	Citric acid (**6**)	1.50	44.30	<0.001
	4	Glutaminsaeure anhydride (**7**)	2.56	0.29	<0.01
	5	Esculetin (**15**)	2.72	0.07	<0.001
	6	Ophiofurospiside L (**17**)	1.17	0.45	<0.05
	7	Ophiopogonin F (**18**)	3.10	0.49	<0.05
	8	(2aS,3aS)-lyciumamide D (**31**)	1.26	0.10	<0.01
	9	5,7,4′-trihydroxy-homoisoflavone (**37**)	3.64	18.81	<0.01
	10	Cixi-ophiopogon C (**42**)	1.05	0.39	<0.05
	11	Ophiopogonin D (**73**)	2.06	3.99	<0.01
	12	Ruscogenin-1-O-Sulfate (**75**)	1.33	0.24	<0.05
	13	Lyso-PE (16:0) (**85**)	2.62	19.23	<0.001
	14	Ophiopogonanone C (**90**)	2.45	11.58	<0.05
LS vs. WS	1	Raffinose (**2**)	4.51	0.46	<0.01
	2	L-Malic acid (**3**)	1.01	9.66	<0.05
	3	N-Fructosyl pyroglutamate (**5**)	2.83	0.22	<0.01
	4	Citric acid (**6**)	1.94	83.90	<0.001
	5	Glutaminsaeure anhydride (**7**)	2.58	0.46	<0.05
	6	Ophiopogonin P (**13**)	2.15	0.41	<0.01
	7	Esculetin (**15**)	2.48	0.20	<0.01
	8	Feruloyltyramine (**16**)	1.12	0.14	<0.01
	9	Ophiofurospiside L (**17**)	1.32	0.36	<0.05
	10	Ophiopogonin F (**18**)	3.80	0.35	<0.05
	11	Ophiofurospiside N (**23**)	2.57	0.13	<0.05
	12	(2aS,3aS)-lyciumamide D (**31**)	1.36	0.10	<0.01
	13	5,7,4′-trihydroxy-homoisoflavone (**37**)	5.07	44.95	<0.01
	14	Lyso-PE (16:0) (**85**)	1.95	18.16	<0.001
	15	Ophiopogonanone C (**90**)	2.45	10.58	<0.05
	16	8-formylophipogonanone B (**92**)	1.38	11.36	<0.05

## Data Availability

The data presented in this study are available in Supplementary Materials. The data that support the findings of this study are available from the corresponding author upon reasonable request.

## Supplementary Materials

The following supporting information can be downloaded at: https://www.mdpi.com/article/10.3390/metabo13020204/s1. Figure S1: Representative compounds of each cluster of MD with different levels of SF in the molecular network. Steroidal saponins (A); Homoisoflavonoids (B); Polysaccharides (C); Others (D); Figure S2: The enlargement of cluster Ⅲ molecular network and possible fragmentation pathway of Raffinose (Compound **2**) from MD in negative ion mode; Figure S3: PCA score plots of HS and WS (A, R^2^X = 0.685), LS and WS (B, R^2^X = 0.711) samples in negative ion mode. Table S1: Identification of the chemical compounds of Ophiopogonis Radix by UPLC-QTOF-MS.
