# Supplementary material for: Characterization and Discrimination of Ophiopogonis Radix with Different Levels of Sulfur Fumigation Based on UPLC-QTOF-MS Combined Molecular Networking with Multivariate Statistical Analysis
Source: Metabolites. 2023 Jan 30;13(2):204. doi: 10.3390/metabo13020204 (PMC9963253; doi:10.3390/metabo13020204)
Supplement: Supplementary file 1 [file metabolites-13-00204-s001.zip › metabolites-2137273-supplementary.pdf]

Supplementary Materials

Characterization and discrimination of Ophiopogonis Radix with different levels of sulfur fumigation based on UPLC-QTOF-MS combined molecular networking with multivariate statistical analysis

Yanhui Lv [1,2,†], Xike Xu [2,†], Yanping Wei [1,2,†], Yunheng Shen [2], Wei Chen [2], Xintong Wei [1,2], Jie Wang [1,2], JiaYun Xin [1,2], Jixiang He [1,*], Xianpeng Zu [2,*]

Supplementary Figure Legends

Figure S1. Representative compounds of each cluster of MD with different levels of SF in the molecular network. Steroidal saponins (A); Homoisoflavonoids (B); Polysaccharides (C); Others (D).

Figure S2. The enlargement of cluster III molecular network and possible fragmentation pathway of Raffinose (Compound **2**) from MD in negative ion mode.

Figure S3. PCA score plots of HS and WS (A, $R^2X = 0.685$), LS and WS (B, $R^2X = 0.711$) samples in negative ion mode.

Supplementary Tables

Table S1. Identification of the chemical compounds of Ophiopogonis Radix by UPLC-QTOF-MS.

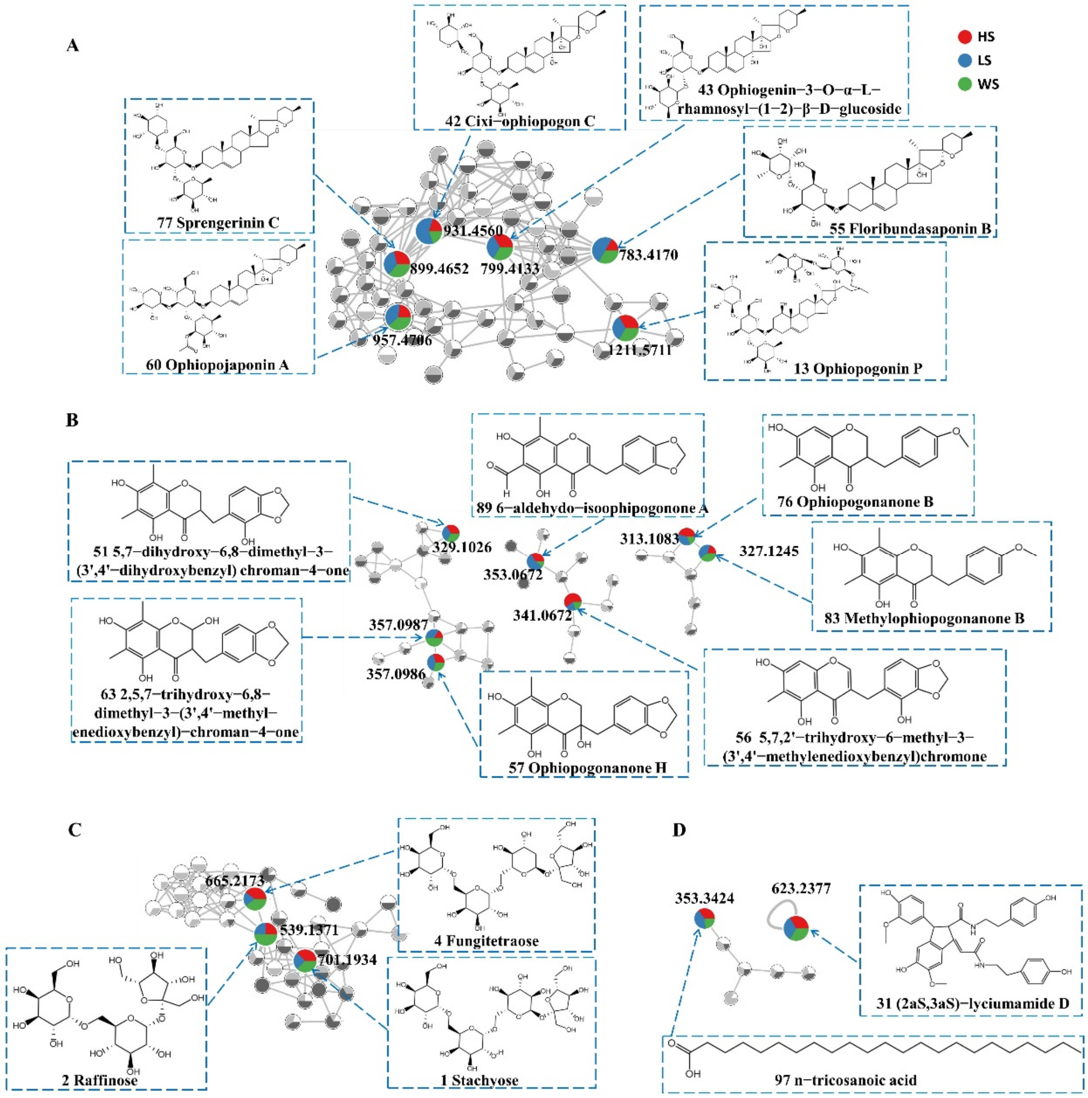

Figure S1. Representative compounds of each cluster of MD with different levels of SF in the molecular network. Steroidal saponins (A); Homoisoflavonoids (B); Polysaccharides (C); Others (D).

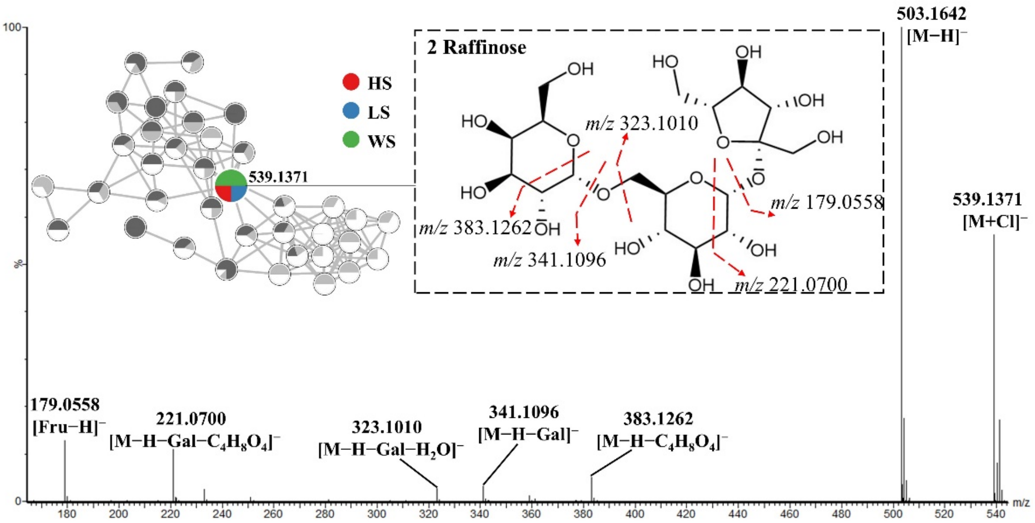

Figure S2. The enlargement of cluster III molecular network and possible fragmentation pathway of Raffinose (Compound **2**) from MD in negative ion mode.

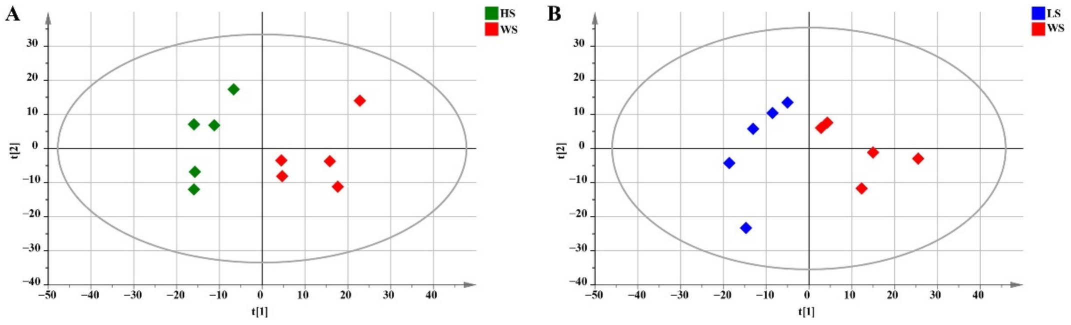

Figure S3. PCA score plots of HS and WS (A, R^2X = 0.685), LS and WS (B, R^2X = 0.711) samples in negative ion mode.

Table S1. Identification of the chemical compounds of Ophiopogonis Radix by UPLC-QTOF-MS.

No.	Rt	Identification	Formula	MS	Error	MS/MS	Classification
**1**	0.60	Stachyose	$C_{24}H_{42}O_{21}[M+Cl]^-$	701.1934	3.9	665.2176;503.1642;485.1540;383.1262;341.1096;221.0700;179.0558	Polysaccharides
**2**	0.62	Raffinose*	$C_{18}H_{32}O_{16}[M+Cl]^-$	539.1371	−1.4	503.1642;383.1262;341.1096;323.1010;221.0700;179.0558	Polysaccharides
**3**	0.72	L-Malic acid*	$C_4H_6O_5[M-H]^-$	133.0144	5.0	115.0013	Organic acids
**4**	0.80	Fungitetraose	$C_{24}H_{42}O_{21}[M-H]^-$	665.2173	4.9	503.1642;485.1529;383.1237;341.1130	Polysaccharides
**5**	0.90	N-Fructosyl pyroglutamate*	$C_{11}H_{17}NO_8[M-H]^-$	290.0876	1.3	200.0562;128.0351	Alkaloids
**6**	0.91	Citric acid*	$C_6H_8O_7[M-H]^-$	191.0187	−2.6	173.0111	Organic acids
**7**	0.93	Glutaminsaeure anhydride*	$C_5H_7NO_3[M-H]^-$	128.0360	5.5	113.0244	Others
**8**	1.68	Vanillic acid	$C_8H_8O_4[M-H]^-$	167.0349	−0.6	152.0128;108.0214	Organic acids
**9**	1.88	3-p-Coumaroylquinic acid or isomer	$C_{16}H_{18}O_8[M-H]^-$	337.0934	3.2	191.0549;163.0390;119.0515	Organic acids
**10**	1.95	Caffeic acid-3-*O*-β-D-glucopyranoside	$C_{15}H_{18}O_9[M-H]^-$	341.0889	3.3	179.0347	Others
**11**	2.00	Ophiopogonin H	$C_{56}H_{92}O_{29}[M+HCOO]^-$	1273.5721	1.1	1227.5712;1095.5277;1077.5171;1065.5161;931.4574;787.4143	Steroidal saponins
**12**	2.00	3-p-Coumaroylquinic acid or isomer	$C_{16}H_{18}O_8[M-H]^-$	337.0934	3.2	191.0549;173.0470;163.0390;119.0515	Organic acids
**13**	2.04	Ophiopogonin P*	$C_{56}H_{92}O_{28}[M-H]^-$	1211.5711	0.7	1079.5300;1065.5139;1049.5146;917.4702;771.4180;609.3683;591.3596	Steroidal saponins
**14**	2.05	Caffeic acid	$C_9H_8O_4[M-H]^-$	179.0352	0.9	135.0469	Organic acids
**15**	2.05	Esculetin*	$C_9H_6O_4[M-H]^-$	177.0193	2.8	133.0295;105.0374	Others
**16**	2.08	Feruloyltyramine*	$C_{18}H_{19}NO_4[M-H]^-$	312.1239	−0.8	297.1052;190.0504;178.0521;148.0540;135.0469	Alkaloids
**17**	2.11	Ophiofurospiside L*	$C_{50}H_{82}O_{23}[M+HCOO]^-$	1095.5238	−1.5	917.4702;903.4650;899.4789;771.4240;609.3683;591.3596	Steroidal saponins
**18**	2.12	Ophiopogonin F*	$C_{56}H_{92}O_{27}[M-H]^-$	1195.5752	0.8	1063.5347;1033.5259;901.4839;755.4238;593.3710;575.3587	Steroidal saponins
**19**	2.14	Ophiofurospiside M	$C_{45}H_{74}O_{19}[M-H]^-$	917.4756	0.5	771.4219;755.4153;609.3683;591.3596	Steroidal saponins

**20**	2.17	3,4-dihydroxy-allylbenzene-4-*O*-β-D-rhamnopyranosyl-(1-6)-β-D-glucopyranoside	$C_{21}H_{30}O_{11}$[M−H]$^{-}$	457.171	−1.1	149.0600	Others
**21**	2.19	Ophiopogonin I'	$C_{51}H_{82}O_{23}$[M−H]$^{-}$	1061.5179	0.5	915.4622;899.4649	Steroidal saponins
**22**	2.21	cixi-ophiopogon B	$C_{50}H_{80}O_{23}$[M−H]$^{-}$	1047.501	−0.7	915.4614;769.3983	Steroidal saponins
**23**	2.24	Ophiofurospiside N*	$C_{50}H_{82}O_{22}$[M−H]$^{-}$	1033.5238	1.3	901.4839;887.4692;755.4153	Steroidal saponins
**24**	2.26	(22S)-cholest-5-ene-1β,3β,16β,22-tetrol 1-*O*-α-L-rhamnopyranosyl-16-*O*-β-D-glucopyranoside	$C_{39}H_{66}O_{13}$[M+HCOO]$^{-}$	787.457	−1.3	741.4453;579.3988;433.3360	Steroidal saponins
**25**	2.27	3,4-dihydroxy-allylbenzene-4-*O*-β-D-glucopyranoside	$C_{15}H_{20}O_{7}$[M−H]$^{-}$	311.1125	−3.5	149.0600	Others
**26**	2.31	Ophiopogonin K'	$C_{56}H_{90}O_{26}$[M+HCOO]$^{-}$	1223.5716	1.1	1177.5662;1031.5078;1015.5108;899.4653;883.4686;737.4107	Steroidal saponins
**27**	2.31	26-*O*-glc-(25R)-5,20(22)-diene-furost-1*β*,3*β*,26-triol-3-*O*-rha-(1-2)-[xyl-(1-4)]-glc	$C_{50}H_{80}O_{22}$[M+HCOO]$^{-}$	1077.5187	−0.8	1031.5035;899.4696;737.4173	Steroidal saponins
**28**	2.38	Borneol-7-*O*-β-D-xylopyranosyl-(1-6)-β-D-glucopyranoside	$C_{21}H_{36}O_{10}$[M−H]$^{-}$	447.223	−1.3	315.1823	Others
**29**	2.44	N-trans-coumaroyltyramine	$C_{17}H_{17}NO_{3}$[M−H]$^{-}$	282.1134	−0.5	119.0498	Alkaloids
**30**	2.52	Cryptomeridiol-11-*O*-β-D-xylopyranosyl-(1-6)-β-D-glucopyranoside	$C_{26}H_{46}O_{1}$[M+HCOO]$^{-}$	579.3039	2.9	533.2940;401.2524	Others
**31**	2.65	(2aS,3aS)-lyciumamide D*	$C_{36}H_{36}N_{2}O_{8}$[M−H]$^{-}$	623.2377	−2.6	460.1738;283.0998	Alkaloids
**32**	2.96	5,6,7-thihydroxy-6-methyl-3-(4'-hydroxybenzyl)	$C_{17}H_{16}O_{5}$[M+HCOO]$^{-}$	345.0989	2.7	139.0397	Homoisoflavonoids

		chroman-4-one					
**33**	3.17	3,5-dihydroxy-7-methoxy-3-(4-hydroxybenzyl) chroman-4-one	$C_{17}H_{16}O_6[M-H]^-$	315.0882	2.6	209.0475;165.0193;139.0397	Homoisoflavonoids
**34**	3.22	Tianshic acid	$C_{18}H_{34}O_5[M-H]^-$	329.2344	3.3	311.2218;293.2126	Organic acids
**35**	3.32	5,7,2',4'-tetrahydroxy-8-methoyl-6-methyl-homoisoflavanone	$C_{18}H_{18}O_7[M-H]^-$	345.0988	2.5	209.0480;181.0498;139.0397	Homoisoflavonoids
**36**	3.32	Ophiopogonone D or isomer	$C_{17}H_{14}O_6[M-H]^-$	313.0713	−1.6	204.0415;191.0368;175.0405;139.0397	Homoisoflavonoids
**37**	3.4	5,7,4'-trihydroxy-homoisoflavone*	$C_{16}H_{12}O_5[M-H]^-$	283.062	2.8	190.0285;177.0163;151.0025	Homoisoflavonoids
**38**	3.41	Ophiopogonin R	$C_{39}H_{62}O_{15}[M+HCOO]^-$	815.4052	−2.2	769.4117;623.3436;461.2914;443.2834	Steroidal saponins
**39**	3.45	Ophiopogonanone G	$C_{17}H_{16}O_6[M-H]^-$	315.087	−1.2	209.0437;193.0521	Homoisoflavonoids
**40**	3.49	Isoophiopogonone A	$C_{18}H_{14}O_6[M-H]^-$	325.0712	−1.8	313.0715;203.0349;191.0349	Homoisoflavonoids
**41**	3.52	Ophiopogonone D or isomer	$C_{17}H_{14}O_6[M-H]^-$	313.0729	3.7	298.0469;204.0415;175.0405	Homoisoflavonoids
**42**	3.65	Cixi-ophiopogon C*	$C_{44}H_{70}O_{18}[M+HCOO]^-$	931.4560	1.7	885.4495;753.4206;607.3493;445.2956	Steroidal saponins
**43**	3.84	Ophiogenin-3-O-α-L-rhamnosyl-(1-2)-β-D-glucoside	$C_{39}H_{62}O_1[M+HCOO]^-$	799.4133	1.4	753.4008;607.3498;445.2959;427.2845	Steroidal saponins
**44**	3.93	Ophiopogonanone E	$C_{19}H_{20}O_7[M-H]^-$	359.1126	−2.8	341.1020;325.0703;205.0495	Homoisoflavonoids
**45**	3.97	Ophiopogonside A	$C_{26}H_{44}O_{10}[M+HCOO]^-$	561.2935	4.3	515.2930;383.2459	Others
**46**	4.02	5,7-dihydroxy-6-methyl-3-(4'-hydroxybenzyl) chromone	$C_{17}H_{14}O_5[M-H]^-$	297.0775	2.3	204.0415;191.0368;165.0190	Homoisoflavonoids
**47**	4.07	Ophiopogonanone F	$C_{20}H_{22}O_7[M-H]^-$	373.1297	1.2	358.1082;222.0580;207.0540;183.0664;168.0431;153.0198	Homoisoflavonoids
**48**	4.17	desmethylisoophiopogonone B	$C_{17}H_{14}O_5[M-H]^-$	297.0777	2.7	191.0344;165.0203;139.0405	Homoisoflavonoids
**49**	4.34	Flavokawain C	$C_{17}H_{16}O_5[M-H]^-$	299.0934	3	193.0511;178.0274;165.0201	Homoisoflavonoids
**50**	4.45	6-aldehydo-	$C_{19}H_{16}O_6[M-H]^-$	339.0866	−2.5	324.0672;295.0612;139.0397	Homoisoflavonoids

		isoophipogonone B					
**51**	4.53	5,7-dihydroxy-6,8-dimethyl-3-(3',4'-dihydroxybenzyl) chroman-4-one	$C_{18}H_{18}O_6$[M−H]$^-$	329.1026	−1.3	206.0583;191.0350	Homoisoflavonoids
**52**	4.64	25-(R)-Ruscogenin-1-*O*-β-D-glucopyranose-(1-2)-β-D-xylopyranosyl-(1-3)-β-D-fucopyranoside	$C_{44}H_{70}O_{17}$[M−H]$^-$	869.4536	−0.5	869.4536;737.4113;719.4024;591.3529	Steroidal saponins
**53**	4.71	5,7,2'-trihydroxy-6,8-dimethyl-3-(3',4'-methylenedioxybenzyl) chromone	$C_{17}H_{14}O_5$[M+HCOO]$^-$	343.0824	0.2	139.0397	Homoisoflavonoids
**54**	4.86	5,7-dihydroxy-6,8-dimethyl-3-(2',6'-methoxybenzyl-4'-dihydroxy) chroman-4-one	$C_{20}H_{22}O_7$[M−H]$^-$	373.1297	1.0	207.0667;192.0419;179.0325	Homoisoflavonoids
**55**	4.93	Floribundasaponin B	$C_{39}H_{62}O_{13}$[M+HCOO]$^-$	783.417	−0.3	737.4173;591.3596	Steroidal saponins
**56**	5.03	5,7,2'-trihydroxy-6-methyl-3-(3',4'-methylenedioxybenzyl) chromone	$C_{18}H_{14}O_7$[M−H]$^-$	341.0672	1.5	204.0415;191.0368;175.0405;139.0397	Homoisoflavonoids
**57**	5.15	Ophiopogonanone H	$C_{19}H_{18}O_7$[M−H]$^-$	357.0986	1.6	342.0782;327.0536;299.0580;153.0560	Homoisoflavonoids
**58**	5.18	5,7,4'-trihydroxy-3'-methoxy-6,8-dimethyhomoisoflavanone	$C_{19}H_{20}O_6$[M−H]$^-$	343.1184	−0.9	207.0657;179.0698;139.0397	Homoisoflavonoids
**59**	5.23	5,3',4'-trihydroxy-7,8-dimethoxy-6-methyl homoisoflavone	$C_{19}H_{20}O_7$[M−H]$^-$	359.1141	1.3	329.0707;237.0764;222.0515;207.0280;153.0210	Homoisoflavonoids
**60**	5.31	Ophiopojaponin A	$C_{46}H_{72}O_{18}$[M+HCOO]$^-$	957.4706	0.5	911.4637;869.4533;851.4395;737.4123	Steroidal saponins
**61**	5.33	5,7-dihydroxy-8-	$C_{19}H_{20}O_7$[M−H]$^-$	359.1146	2.6	344.0909;223.0604;208.0379;169.0507;15	Homoisoflavonoids

		methoxy-6-methyl-3-(2'-hydroxy-4'-methoxybenzyl chroman-4-one)				4.0271	
**62**	5.45	Ophiopogonone E	$C_{19}H_{18}O_7$[M−H]$^-$	357.099	2.9	339.0875;327.0496;221.0441;207.0298;179.0353;169.0502	Homoisoflavonoids
**63**	5.67	2,5,7-trihydroxy-6,8-dimethyl-3-(3',4'-methyl-enedioxybenzyl)-chroman-4-one	$C_{19}H_{18}O_7$[M−H]$^-$	357.0987	2.1	325.0722;163.0042;153.0556	Homoisoflavonoids
**64**	5.67	Ophiopojaponin E	$C_{38}H_{60}O_{13}$[M+HCOO]$^-$	769.4022	0.8	723.3972;591.3544	Steroidal saponins
**65**	5.8	Pennogenin -3-O-Ac-(1-2)-rha-(1-2)-[xyl-(1-4)]-glc	$C_{46}H_{72}O_{18}$[M+HCOO]$^-$	957.471	1.0	911.4642;869.4528;851.4478;737.4109	Steroidal saponins
**66**	5.82	5,7-dihydroxy-8-methoxy-6-methyl-3-(2'-hydroxy-4'-methoxybenzyl chroman-4-one)	$C_{19}H_{20}O_6$[M−H]$^-$	343.1193	1.6	207.0657;153.0556	Homoisoflavonoids
**67**	5.94	2'-hydroxymethylophiopogonone A	$C_{19}H_{16}O_7$[M−H]$^-$	355.083	2.0	218.0609;205.0534;189.0561	Homoisoflavonoids
**68**	6.02	5,3'-dihydroxy-7,4'-dimethoxy-6-methyl homoisoflavone	$C_{19}H_{18}O_6$[M−H]$^-$	341.1035	1.4	217.0501;205.0490;189.0562	Homoisoflavonoids
**69**	6.1	Ruscogenin 3-*O*-Ac-(1-4)-rha-(1-2)-glc	$C_{41}H_{64}O_{14}$[M+HCOO]$^-$	825.4286	1.0	779.4254;737.4144	Steroidal saponins
**70**	6.2	Ophiopogonin P''	$C_{41}H_{64}O_{14}$[M+HCOO]$^-$	825.4278	0	779.4241;737.4096	Steroidal saponins
**71**	6.3	Ophiopogonone A	$C_{18}H_{14}O_6$[M−H]$^-$	325.0719	0.3	297.0751;204.0415;191.0368;165.0190	Homoisoflavonoids
**72**	6.38	Isoophiopogonanone A	$C_{18}H_{16}O_6$[M−H]$^-$	327.0879	1.6	193.0521;165.0583	Homoisoflavonoids
**73**	6.46	Ophiopogonin D*	$C_{44}H_{70}O_{16}$[M+HCOO]$^-$	899.4644	−0.2	853.4549;721.4142;575.3587	Steroidal saponins
**74**	6.53	Ophiopogonanone A	$C_{18}H_{16}O_6$[M−H]$^-$	327.0879	1.5	193.0521;164.0499;136.0180	Homoisoflavonoids
**75**	6.57	Ruscogenin-1-*O*-	$C_{27}H_{42}O_7S$[M−H]$^-$	509.2592	2.6	509.2567;96.9587	Others

		Sulfate*					
**76**	6.77	Ophiopogonanone B	$C_{18}H_{18}O_5$[M−H]⁻	313.1083	0.5	192.0422;164.0476;136.0159	Homoisoflavonoids
**77**	6.82	Sprengerinin C	$C_{44}H_{70}O_{16}$[M+HCOO]⁻	899.4652	0.7	853.4587;721.4168;575.3629	Steroidal saponins
**78**	7.26	Methylophiopogonone A	$C_{19}H_{16}O_6$[M−H]⁻	339.0875	0.3	218.0544;205.0516;179.0340	Homoisoflavonoids
**79**	7.41	Ophiopogonin P'	$C_{46}H_{72}O_{17}$[M+HCOO]⁻	941.4746	−0.6	895.4675;853.4489;835.4471;721.4147	Steroidal saponins
**80**	7.48	25-(S)-Ruscogenin-1-*O*-β-D-fucopyranosyl-3-O-α-L-rhamnopyranoside	$C_{39}H_{62}O_{12}$[M+HCOO]⁻	767.4231	1.0	721.4178;575.3591	Steroidal saponins
**81**	7.58	Methylophiopogonanone A	$C_{19}H_{18}O_6$[M−H]⁻	341.1035	1.4	207.0657;178.0635;149.0227	Homoisoflavonoids
**82**	7.8	5,7-trihydroxy-6,8-dimethyl-3-(2'-hydroxy-3',4'-methylenedioxybenzyl) chromone	$C_{19}H_{18}O_7$[M−H]⁻	357.0982	0.7	329.1024;208.0393;165.0186	Homoisoflavonoids
**83**	7.9	Methylophiopogonanone B	$C_{19}H_{20}O_5$[M−H]⁻	327.1245	2.1	206.0581;178.0637;163.0403;149.0244;135.0451	Homoisoflavonoids
**84**	8.22	Ophiopogonin Q'	$C_{46}H_{72}O_{17}$[M+HCOO]⁻	941.4763	1.2	895.4691;853.4586;835.4501;721.4140	Steroidal saponins
**85**	8.35	1-hexadecanoyl-sn-glycero-3-phosphoethanolamine*	$C_{21}H_{44}NO_7P$[M−H]⁻	452.2757	−4.4	255.2327;196.0369	Others
**86**	8.48	Unknow	—[M−H]⁻	571.2905	3.8	255.2327;241.0129;152.0040	Others
**87**	8.97	Ophiopogonin A	$C_{41}H_{64}O_{13}$[M+HCOO]⁻	809.4324	−0.6	763.4271;721.4142;703.4053	Steroidal saponins
**88**	9.55	(25S)-ruscogenin1-*O*-2,3-O-diacetyl-α-L-rhamnopyranosyl-(1-2)-[β-D-xylopyranosyl-(1-3)]-β-D-fucopyranoside	$C_{48}H_{74}O_{18}$[M+HCOO]⁻	983.4842	−1.0	937.4797;895.4667;853.4578;835.44880;721.4136;707.4008	Steroidal saponins
**89**	9.56	6-aldehydo-isoophipogonone A	$C_{19}H_{14}O_7$[M−H]⁻	353.0672	1.4	325.0708;297.0755;191.0344	Homoisoflavonoids
**90**	9.87	Ophiopogonanone C*	$C_{19}H_{16}O_7$[M−H]⁻	355.0828	1.5	327.0853;193.0157	Homoisoflavonoids
**91**	9.93	8-formylophipogonone	$C_{19}H_{16}O_6$[M−H]⁻	339.0874	0	323.0556;311.0927;295.0614;189.0200	Homoisoflavonoids

		B					
**92**	10.29	8-formylophipogonanone B*	$C_{19}H_{18}O_6[M-H]^-$	341.103	−0.3	313.1079;221.0375;192.0418;164.0479	Homoisoflavonoids
**93**	12.94	5,7-dihydroxy-8-formyl-3-(4'-methoxybenzyl) chromone	$C_{18}H_{14}O_6[M-H]^-$	325.0721	1.0	191.0368	Homoisoflavonoids
**94**	13.08	Ophiopogonside A isomer	$C_{26}H_{44}O_{10}[M+HCOO]^-$	561.2935	4.3	205.0534	Others
**95**	15.49	[1-hexadecanoyloxy-3-[hydroxy-(2,3,4,5,6-pentahydroxycyclohexyl) oxyphosphoryl] oxypropan-2-yl] (9Z,12Z)-octadeca-9,12-dienoate	$C_{43}H_{79}O_{13}P[M-H]^-$	833.5176	0.5	553.2772;391.2224;279.2321;255.2323	Others
**96**	16.95	stigmasterol-beta-d-glucoside	$C_{35}H_{58}O_6[M+HCOO]^-$	619.4219	0.6	−	Others
**97**	20.23	n-tricosanoic acid	$C_{23}H_{46}O_2[M-H]^-$	353.3424	−0.2	−	Organic acids
**98**	20.84	β-sitosterol-β-D-glucopyranoside	$C_{35}H_{60}O_6[M+HCOO]^-$	621.4373	0.2	−	Others

−: No related references found.

*: Chemical markers selected by multivariate statistical analysis.
